# Supplementary material for: Patterns of Intron Gain and Loss in Fungi
Source: PLoS Biol. 2004 Nov 30;2(12):e422. doi: 10.1371/journal.pbio.0020422 (PMC532390; doi:10.1371/journal.pbio.0020422)
Supplement: Table S1 — Also available at http://genes.mit.edu/NielsenEtAl/. (4.3 MB ZIP). [file pbio.0020422.st001.zip › NielsenEtAl/html/891.html]

AN4159.1.NCU06724.1.MG06888.1.FG10264.1


```
 CLUSTAL W (1.82) Multiple Sequence Alignments - Introns Inserted


Sequence 1: NCU06724.1	345 aa
Sequence 2: MG06888.1	346 aa
Sequence 3: FG10264.1	354 aa
Sequence 4: AN4159.1	345 aa
Alignment Length: 360 aa
Number Identitical Residues: 203 aa
Alignment Score (without introns) 10265


MG06888.1 	-~----------MAKYMSLDQKGSIMAEYVWIDSEGGVRSKSR0TL--KEQKYEPKDLPT
NCU06724.1	-~--------------MRLPQNGQLIAEYIWIDSEGGTRSKSR0TLPEKESQYLPEELPI
FG10264.1 	M0AAPITSRTETLQKYLKLDQKGMIMAEYVWVDAEGGTRSKSR0TLP--EKDYKPEDLPV
AN4159.1  	-~-----------MKYMSLDQRGSVMAEYIWIDAHGGTRSKTK0TLS--KAPSSVDELPE
          	              . : * *.* ::***:*:*:.**.***:: **.  :     .:** 

MG06888.1 	WNFDGSSTGQAGGDNSDVFLK~PVAVYPDPFRGEPNIIVLAECWDSDGTPNKFNHRHEAA
NCU06724.1	WNFDGSSTGQAPGENSDVYLK0PVAVFPDPFRGSPNILVLSECWNADGTPNKFNHRHEAA
FG10264.1 	WNFDGSSTKQAPGENSDVYLR~PCAVYPDPFRGSPNIIVLAECYDSDGTPNKYNFRHDCV
AN4159.1  	WNFDGSSTAQAPGDNSDVYLR~PVAMYPDPFRRGDNILVLCETWDSDGSPNKFNYRHDCA
          	******** ** *:****:*: * *::*****   **:**.* :::**:***:*.**:..

MG06888.1 	KLMEAHAEHEPWFGLEQEYTLLNLEDRPFGWPKNGFPAP~QGPYYCGVGAGKVVQRDIVE
NCU06724.1	KLMEAHADQVPWFGLEQEYTLLDINDRPYGWPRNGFPAP~QGPYYCGVGTGKVVQRDIVE
FG10264.1 	KVMKTYADEEPWFGLEQEYTLLGSDDRPYGWPAGGFPAP2QGEYYCGVGTGKVVQRDIVE
AN4159.1  	RLMETHAKEEFWFGLEQEYTLLGPDGWPYGWPKGGFPGA~QGPYYCGVGTGKVYCRDIVE
          	::*:::*..  ***********. :. *:*** .***.. ** ******:***  *****

MG06888.1 	AHYKACLY~AGVKISGTNAEVMPAQWEFQVGPCEGIEM1GDHLSIARFLLHRVAEEFGAK
NCU06724.1	AHYKCCLY~AGVKISGTNAEVMPAQWEFQVGPCDGIEM~GDHLWLARFLLHRVSEEFGAK
FG10264.1 	AHYKACLY1AGIQISGTNAEVMPAQWEYQVGPCTGIEM~GDQLWVSRFFLHRVAEEFGAK
AN4159.1  	AHYRACLY~AGVKISGINAEVMPSQWEYQVGPCHGIEM~GDHLWISRFLLHRVAEEFGVK
          	***:.*** **::*** ******:***:***** **** **:* ::**:****:****.*

MG06888.1 	VSFDPKPIPGDWNGAGLHS~NFSTKEMRVDGGMKHIEAAIKKLEGRHKEHIAVYGEGNEK
NCU06724.1	VSFDPKPIPGDWNGAGLHT~NFSTENMRKEGGMKYIEDAIKKLEARHKEHIAVYGEGNDK
FG10264.1 	VSLHPKPIAGDWNGAGLHS0NFSTKAMREEGGMKVIEEALKKLEPHHVECIAEYGEDNDL
AN4159.1  	ISFDPKPIKGDWNGAGLHT~NVSTTSTRAEGGIKAIESYMKKLEARHVEHIAVYGEGNEE
          	:*:.**** *********: *.**   * :**:* **  :**** :* * ** ***.*: 

MG06888.1 	RLTGSHETGSIEEFSYGIANRGASIRIPRECAAKGYGYFEDRRPASNADPYQ0ITGIIME
NCU06724.1	RLTGRHETGSIDSFTYGVANRGASIRIPRECGAKGYGYFEDRRPASNADPYQ~ITGIIME
FG10264.1 	RLTGRHETGSIDSFSWGVANRGTSIRVPRETAAKGYGYFEDRRPASNADPYR~VTKVLLQ
AN4159.1  	RLTGRHETGNIDKFSYGVADRGGSIRIPRQVAKDGKGYFEDRRPASNADPYQ~ITGIIAE
          	**** ****.*:.*::*:*:** ***:**: . .* ***************: :* :: :

MG06888.1 	TCFGAVN-
NCU06724.1	TCFGAVSE
FG10264.1 	FSME----
AN4159.1  	TVSFSNR-
          	    :
```
